# Characterization of prion strains and peripheral prion infectivity patterns in E200K genetic CJD patients

**DOI:** 10.1007/s00401-025-02903-5

**Published:** 2025-06-16

**Authors:** Tomás Barrio, Jean-Yves Douet, Dana Žáková, Hasier Eraña, Alvina Huor, Hervé Cassard, Oihane Alzuguren, Séverine Lugan, Naïma Aron, Patrice Péran, Joaquín Castilla, Olivier Andréoletti

**Affiliations:** 1 UMR INRAE-ENVT 1225, Interactions Hôtes-Agents Pathogènes, Ecole Nationale Vétérinaire de Toulouse, 23 Chemin des Capelles, 31076 Toulouse, France; 2 Department of Prion Diseases, Slovak Medical University, Bratislava, Slovakia; 3 Center for Cooperative Research in Biosciences (CIC bioGUNE), Basque Research and Technology Alliance (BRTA), Derio, Bizkaia Spain; 4 ATLAS Molecular Pharma S. L., Derio, Bizkaia Spain; 5 Centro de Investigación Biomédica en Red de Enfermedades infecciosas (CIBERINFEC), Carlos III National Health Institute, Madrid, Spain; 6 UMR INSERM-UT3 1214, Toulouse NeuroImaging Center (ToNIC), CHU Purpan–Pavillon BAUDOT, Toulouse, France; 7 IKERBASQUE, Basque Foundation for Science, Bilbao, Spain

**Keywords:** Prion disease, Genetic CJD

## Abstract

The mutation E200K in the prion protein gene (*PRNP*) is the most common variant in genetic Creutzfeldt–Jakob disease (gCJD). The clinical and pathological features observed in patients with E200K gCJD led to the hypothesis that the prion strains responsible for this form of the disease may be related to those involved in sporadic CJD (sCJD). In this study, we characterized the prion strains responsible for E200K gCJD cases from Slovakia (*n* = 12), Spain (*n* = 9), and France (*n* = 3) using transgenic mouse models expressing human prion protein (PrP). The cohort included patients with various *PRNP* genotypes: E200K-Met_129_/Met_129_, E200K-Met_129_/E200K-Met_129_, E200K-Met_129_/Val_129_, and E200K-Val_129_/Val_129_. Prion strain characterization revealed that the strains isolated from E200K gCJD cases corresponded to the two most common strains identified in sCJD cases: M1^CJD^ and V2^CJD^. Depending on the individual, these strains were either present as pure M1^CJD^ or V2^CJD^, or as a mixture of both (M1^CJD^ + V2^CJD^). Additionally, peripheral tissues from E200K-Met_129_/Met_129_ patients (*n* = 4) and one E200K-Met_129_/Val_129_ case were analyzed for prion infectivity and seeding activity. Similar to sCJD patients, low but detectable levels of prions were found in various peripheral tissues of E200K gCJD cases. Overall, our findings suggest that the prion strains and their distribution in the body are highly similar between E200K gCJD and sCJD patients. These similarities indicate that individuals carrying the E200K mutation may serve as a valuable model for understanding CJD pathogenesis during the preclinical phase of the disease.

**Supplementary Information:**

The online version contains supplementary material available at 10.1007/s00401-025-02903-5.

## Introduction

Prion diseases, also known as transmissible spongiform encephalopathies (TSE), are a group of fatal neurodegenerative disorders that naturally occur in humans and various mammalian species. These diseases are characterized by the accumulation of a misfolded form of the normal cellular prion protein (PrP^C^) in the central nervous system (CNS) [34]. The misfolded protein, commonly referred to as PrP^Sc^, is thought to be the major, if not the only, component of the infectious agents, or prions, responsible for these disorders [43].

Research into sheep scrapie, a prion disease in animals, revealed distinct prion strains during serial passage in mice via intracerebral inoculation, identified based on biological properties that stabilize upon successive passages [17]. These properties include incubation periods and neuropathological features, particularly the distribution and severity of spongiform changes in the brain. In the absence of nucleic acids in the prion agent, these strain variations are attributed to conformational differences in PrP^Sc^ that are self-propagating [43].

Human prion diseases occur in sporadic, genetic, and acquired forms, with sporadic Creutzfeldt–Jakob disease (sCJD) being the most common, presenting worldwide at a consistent annual incidence of 1–2 cases per million [54]. Genetic Creutzfeldt–Jakob disease (gCJD) is associated with mutations in the *PRNP* gene, which occur in approximately 10–15% of all CJD cases. The most frequent mutation is E200K (a substitution of glutamine with lysine at codon 200), responsible for disease clusters in Slovakia, Israel, Spain, Italy, and Chile. The E200K mutation follows an autosomal-dominant inheritance pattern and has a penetrance ranging between 60 and 100%, depending on the population studied [46]. E200K gCJD typically manifests at a younger age than sCJD (60.4 vs 68) [26, 32].

The clinico-pathological phenotype of human prion diseases is highly variable and encompass a wide range of histotypes that correlate with clinical and molecular parameters. In sCJD, this variability is strongly influenced by a common polymorphism at codon 129 of *PRNP*, encoding either methionine (M) or valine (V), resulting in three possible genotypes: MM, MV, and VV. Another critical factor is the isoform of the protease-resistant PrP^Sc^ core (PrP^res^), detectable by Western blot in brain tissue. PrP^res^ exists in two major isoforms in sCJD cases, Type 1 (21 kDa) and Type 2 (19 kDa), and these isoforms combine with codon 129 genotypes to form six distinct sCJD subtypes (MM1, MV1, VV1, MM2, MV2, and VV2) [40]. In up to 35% of sCJD patients, both Type 1 and Type 2 PrP^res^ can co-exist in the same or different brain regions [45, 49], suggesting that these subtypes might be associated to different prion strains. Experimental transmission studies in transgenic mice have revealed at least five sCJD prion strains [6, 10–12, 23, 25].

Despite this complexity, two strains, M1^CJD^ and V2^CJD^, account for the most frequent sCJD forms. M1^CJD^ is typically found in MM/MV1 patients, while V2^CJD^ is predominant in VV/MV2 cases [6, 11, 15]. Both strains can co-exist in the brain of a single patient [10].

In genetic CJD, most cases can be classified into six major histo-molecular subtypes that largely mirror those of sCJD, based on the combination of protease-resistant PrP^Sc^ type and the *PRNP* codon 129 genotype, with individual mutations being associated with distinctive histopathological features—for example, the E200K mutation has been linked with thickened synaptic and plaque-like PrP^Sc^ deposits. For a comprehensive review on the subject, see [4, 27].

Clinically, E200K-Met129 CJD patients resemble MM/MV1 sCJD cases, while E200K-Val129 CJD cases resemble MV/VV2 sCJD [9, 20]. These observations suggest that prion strains involved in E200K gCJD might be similar or identical to those in sCJD.

This study tests that hypothesis by conducting experimental transmissions of brain homogenates from 24 E200K gCJD patients from France (*n* = 3), Slovakia (*n* = 12), and Spain (*n* = 9) into human-PrP-expressing transgenic mice (tgHu), utilizing a well-established methodology for prion strain identification [10, 15]. Control cases from France and the United Kingdom, as well as cloned (pure) human prion strains M1^CJD^ and V2^CJD^, were used for comparison. This is the first study to define and compare prion strain characteristics in E200K gCJD cases with sCJD prion strains, identifying M1^CJD^, V2^CJD^, or a mixture of both in E200K cases.

Furthermore, we examined prion accumulation in peripheral tissues from a subset of E200K gCJD patients. Similar to sCJD patients, prion infectivity and seeding activity were confirmed in these tissues using both tgHu bioassays and in vitro amplification by real-time quaking-induced conversion (RT-QuIC). In all cases, the prion strain recovered from peripheral tissues matched that found in the brain.

The strong similarities between E200K gCJD and sCJD, in both prion strain type and tissue distribution, highlight the potential of longitudinal studies in E200K mutation carriers to advance understanding of prion disease pathogenesis, especially during the preclinical phase.

## Materials and methods

### Ethical statement

All animal experiments were conducted in full compliance with institutional and national regulations, adhering to both French national guidelines and European Union Directives 86/609/EEC and 2010/63/EU for the care and use of laboratory animals. The experimental protocols were reviewed and approved by the Committee on the Ethics of Animal Experiments at the authors'institutions, INRAE Toulouse/ENVT (Permit Number: 01734.01).

Regarding the use of human CJD samples, informed consent for research purposes was obtained from all patients or their legal representatives. The human tissue material used in this study received appropriate ethical approvals and was handled in compliance with all relevant legal and ethical standards.

France: Human brain samples were provided by the Brain Bank of CHU Toulouse. Prior to distribution, these samples were pseudo-anonymized to ensure patient confidentiality in accordance with ethical guidelines.

Spain: the human samples used in this research were provided by the biobank of the Hospital Universitario Fundación Alcorcón (HUFA), with the approval of the local Bioethics Committee (CBBA-CBG), under registry number P-CBG-CBBA-0618, and in accordance with the 1964 Helsinki declaration and its later amendments or comparable ethical standards.

Slovakia: The local ethics committee (Slovak Medical University) approved the study under ethical approval number SMU2017. All study procedures have been performed in accordance with the ethical standards in the Declaration of Helsinki and its later amendments.

UK: Human brain samples were obtained from the National CJD Research & Surveillance Unit Brain and Tissue Bank in Edinburgh, UK, which is part of the MRC Edinburgh Brain Bank. For the purposes of this study, samples were pseudo-anonymized using a Brain Bank reference number. All UK cases had informed consent for research and their supply and use in this study was covered by Ethics Approval (LREC 2000/4/157: National Creutzfeldt–Jakob disease tissue bank: acquisition and use of autopsy material for research on human transmissible spongiform encephalopathies, Professor James Ironside, amended date: 9th October 2007).

### E200K gCJD cases

A total of 24 E200K gCJD cases with available frozen cerebral cortex tissue (200 to 500 mg) were included in this study. These patients originated from France (3), Slovakia (12), and Spain (9). In each case, the full PRNP coding sequence was analyzed to determine the *PRNP* haplotype, following the methods described by Arsac et al. [2] and Barillet et al. [5] (Table 1).

**Table 1:** Transmission of E200K gCJD isolates (10% frontal cortex homogenates) into mice expressing the human PrP (methionine or valine at codon 129)

Isolates				TgMet						TgVal					
				Passage 1			Passage 2			Passage 1			Passage 2		
Case	Genotype	Type	Orig	n/no	Incub (mean ± SD)	PrPres type	n/no	Incub (mean ± SD)	PrPres type	n/no	Incub (mean ± SD)	PrPres type	n/no	Incub (mean ± SD)	PrPres type
1	E200K-Met_129_/Met_129_	1	Fr	6/6	215 ± 18	1	6/6	214 ± 10	1	6/6	323 ± 5	1	6/6	269 ± 11	1
2		1	Slov	6/6	194 ± 9	1	6/6	200 ± 11	1	6/6	267 ± 13	1	6/6	272 ± 6	1
3		1	Slov	6/6	194 ± 8	1	6/6	193 ± 8	1	6/6	298 ± 7	1	6/6	279 ± 10	1
4		1	Slov	6/6	199 ± 24	1	6/6	208 ± 7	1	6/6	319 ± 5	1	6/6	272 ± 17	1
5		1	Slov	6/6	193 ± 9	1	6/6	204 ± 11	1	6/6	270 ± 12	1	6/6	266 ± 6	1
6		1	Slov	6/6	205 ± 4	1	6/6	202 ± 7	1	ND			ND		
7		1	Slov	5/5	232 ± 6	1	6/6	209 ± 9	1	ND			ND		
8		1	Sp	6/6	218 ± 13	1	ND			6/6	303 ± 15	1	ND		
9		1	Sp	6/6	212 ± 21	1	ND			6/6	293 ± 7	1	ND		
10		1	Sp	6/6	213 ± 11	1	6/6	204 ± 8	1	6/6	277 ± 20	1	6/6	274 ± 16	1
11		1	Sp	6/6	211 ± 9	1	6/6	201 ± 6	1	6/6	287 ± 7	1	6/6	268 ± 9	1
12		1	Sp	6/6	228 ± 16	1	6/6	211 ± 12	1	6/6	310 ± 10	1	6/6	284 ± 14	1
13		1	Sp	6/6	241 ± 16	1	ND			6/6	342 ± 109	1	ND		
14		1	Fr	6/6	227 ± 18	1	ND			6/6	300 ± 24	1	ND		
15	E200K-Met_129_/E200K-Met_129_	1	Slov	6/6	204 ± 12	1	6/6	196 ± 5	1	6/6	296 ± 9	1	6/6	267 ± 5	1
16	E200K-Met_129_/E200K Met_129_ E200K-Met_129_/Val_129_	1	Fr	6/6	385 ± 96	1	6/6	190 ± 16	1	6/6	203 ± 16	2	6/6	178 ± 4	2
17		2	Slov	6/6	515 ± 45	1	6/6	513 ± 14	1	6/6	227 ± 9	2	6/6	174 ± 13	2
18		1 + 2	Slov	6/6	192 ± 6	1	6/6	198 ± 7	1	6/6	277 ± 15	1	6/6	260 ± 25	1
19		1	Slov	6/6	181 ± 7	1	6/6	201 ± 10	1	6/6	302 ± 2	1	6/6	263 ± 7	1
20		1	Slov	6/6	187 ± 7	1	6/6	214 ± 5	1	6/6	259 ± 8	1	6/6	272 ± 8	1
21		2	Slov	6/6	525 ± 67	1	6/6	559 ± 42	1	6/6	213 ± 13	2	6/6	179 ± 10	2
22		1 + 2	Sp	6/6	599 ± 41	1	6/6	528 ± 21	1	6/6	212 ± 12	2	6/6	184 ± 8	2
23		1 + 2	Sp	6/6	620 ± 33	1	ND			6/6	233 ± 8	2	6/6	176 ± 6	2
24	E200K-Val_129_/Val_129_	2	Sp	5/5	587 ± 38	1	6/6	495 ± 29	1	6/6	217 ± 17	2	6/6	182 ± 6	2

Transgenic mice that express Met_129_ (tgMet) or Val_129_ (tgVal) human PrP were inoculated intracerebrally (20µL per mouse) with 24 E200K Creutzfeldt–Jakob (CJD) brain tissue homogenates from patients originating (orig.) from three different countries: France (Fr), Slovakia (Slov), or Spain (Sp). The E200K gCJD patients displayed different *PRNP* genotypes at codon 129 (MM: homozygous Met_129_, VV: homozygous Val_129_, MV: heterozygous Met/Val_129_) and PrP^res^ Western blot isoforms (type 1, type 2 or type 1 + 2). In all cases except case 24, E200K mutation was associated to the Met_129_ polymorphism. Case 15 displayed the E200K mutation on both *PRNP* alleles.

After the first passage, brain tissues from clinically affected mice were pooled and used for a second passage in the same line. The PrP^res^ WB isoforms (type 1 or type 2) identified in mouse brains are reported for each two passages. Incubation periods (time to death in days) are shown as mean ± standard deviation (SD). n/n0: number of diseased/number of inoculated mice. *ND* not done

### Tissue homogenate preparation

For each E200K gCJD case, approximately 175 ± 20 mg of frozen brain tissue was homogenized in 5% glucose solution, diluted in distilled water, and adjusted to a 10% (w/v) homogenate using a TeSeE™ Process 48™ homogenizer (Bio-Rad). This homogenate was used for subsequent bioassays and biochemical analyses.

### Transgenic mouse lines

Two transgenic mouse lines were used in this study, collectively referred as tgHu: tg340 (tgMet) and tg361 (tgVal), which express human prion protein (PrP) with methionine or valine at codon 129, respectively, at levels approximately fourfold those of human brain, in a PrP knockout (PrP-KO) background. Both lines were generated as previously described [11, 39], and are homozygous for the human *PRNP* gene. These models are widely used for prion strain characterization due to their ability to propagate human prion strains in a consistent and reproducible manner.

### Mouse bioassays

Female mice, 6–10 weeks old, were anesthetized and inoculated with 2 mg of tissue equivalent (20 µL of a 10% cerebral or cerebellar cortex or peripheral tissue homogenate) into the right parietal lobe using a 25-gauge disposable hypodermic needle. Mice were observed daily for clinical signs of prion disease and were assessed weekly by qualified veterinarians. Neurological symptoms, such as tremor, ataxia, difficulty righting from a supine position, tail rigidity, kyphosis, paralysis of the lower limbs, or bradykinesia, were used to confirm prion disease diagnosis [38].

Mice exhibiting three or more progressive neurological signs were euthanized, and their brains were harvested. Half of the brain was fixed in 10% formol saline, while the other half was frozen at − 20 °C for biochemical analysis. For animals found dead without prior signs of prion disease, brain tissue was collected and frozen. The incubation period was calculated as the mean survival time (in days post-inoculation, dpi) of symptomatic mice confirmed to have prion disease via PrP^res^ detection, with corresponding standard deviations.

### Abnormal PrP detection by western blot

To detect the presence of abnormal, protease-resistant prion protein (PrP^res^), western blot analysis was performed on brain homogenates. Samples were subjected to proteinase K (PK) digestion followed by western blotting, as previously described [28]. Immunodetection was carried on PVDF membranes out using the monoclonal primary antibody Sha31 (1 µg/mL), which recognizes amino acids 145–152 (YEDRYYRE) of PrP, and an anti-mouse HRP-conjugated secondary antibody (Biorad) [16]. This method allowed the identification of distinct PrP^res^ isoforms associated with different prion strains.

### Vacuolar lesion profiles

To assess neuropathological changes, standardized vacuolar lesion profiles were established from Hematoxylin–Eosin (H&E)-stained, paraffin-embedded brain tissue sections, as previously described [17, 18]. Lesion profiling was performed on 3–6 animals per group, and the data were used to compare the extent and distribution of spongiform changes across different brain regions in mice inoculated with various prion strains. These profiles are a key component in prion strain typing and phenotyping.

### Real-time quaking-induced conversion (RT-QuIC)

RT-QuIC assays were performed as previously described [52], utilizing recombinant truncated (aa 90–231) Syrian hamster prion protein (rPrP) as the substrate. Brain or tissue homogenates (10%) were serially diluted in phosphate-buffered saline (PBS) containing 0.1% SDS and 1X N-2 supplement (Gibco). For each serial dilution, 2 µL was used to seed individual wells. Dilutions were tested in quadruplicate. The assay plates were subjected to alternating cycles of shaking (700 rpm, double orbital) and incubation at 45 °C for a total duration of 50 h. Thioflavin T (ThT) fluorescence was recorded every 45 min using 450 nm excitation and 480 nm emission wavelengths. Wells exhibiting an increase in ThT fluorescence above the threshold (defined as the mean fluorescence at t = 0 plus three standard deviations) within the 50-h period were classified as positive. The number of positive and negative replicates for each dilution was used to estimate the seeding activity (SA) titers. Titers were calculated using the Spearman–Kärber method, or the Poisson probabilistic model when fewer than 100% positive reactions were observed at the initial dilution [8].

### Reference M1 and V2 CJD strains

Successive 1/10 dilutions of 10% brain homogenate (frontal cortex) from a sporadic CJD (sCJD) MM1 case (case 1) and a sporadic CJD VV2 case (case 6) were inoculated intracerebrally into transgenic mice expressing human PrP Met129 (tgMet, n = 6) and human PrP Val129 (tgVal, *n* = 6). These data were initially presented in a previous study [21]. The brains from the last PrP^Sc^-positive MM1 tgMet mice and VV2 tgVal mice (from the highest dilution groups) were pooled and used to re-inoculate additional groups of tgMet (*n* = 12) and tgVal (*n* = 12) mice. The brains of these tgMet and tgVal animals were subsequently pooled to create two stocks of reference material, designated as M1^CJD^ and V2^CJD^, which served as reference strains for comparison in this study.

### Data availability

The authors confirm that all relevant data supporting the findings of this study are included within the article and its supplementary materials. Further inquiries can be directed to the corresponding author for additional information.

## Results

A total of 24 E200K gCJD cases were analyzed, originating from France (*n* = 3), Slovakia (*n* = 12), and Spain (*n* = 9). The cohort included individuals with *PRNP* E200K-Met_129_/Met_129_ (*n* = 14), E200K-Met_129_/E200K-Met_129_ (*n* = 1), E200K-Met_129_/Val_129_ (*n* = 8), and E200K-Val_129_/Val_129_ (*n* = 1) genotypes (Table 1 and Supplementary Table S1).

**Fig. 1:**
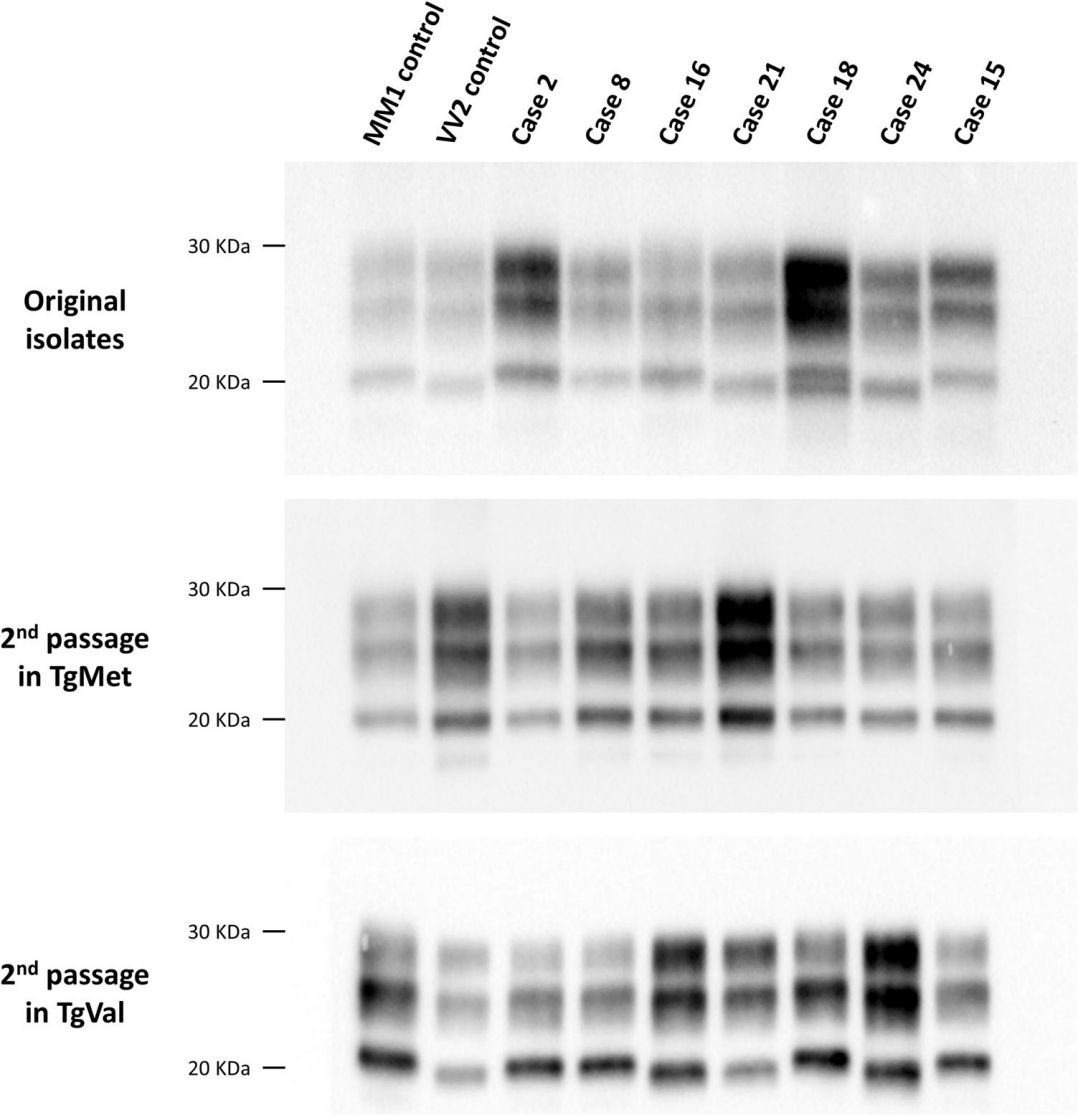
PrP^res^ western blot profiles in original E200K gCJD isolates and E200K gCJD-inoculated human PrP-expressing mice. Western blot analysis of proteinase K-resistant prion protein (PrP^res^) profiles was performed on brain homogenates from both the original E200K gCJD patient isolates and transgenic mice expressing human PrP with Met_129_ (tgMet) or Val_129_ (tgVal) at codon 129. Mice were intracerebrally inoculated with 20 µL of 10% brain homogenate from E200K gCJD patients (n = 6 per group). Two serial passages were carried out in each mouse line (details in Table 1). The PrP^res^ isoform (type 1 or type 2) was identified via SDS-PAGE and Western blot using the anti-PrP monoclonal antibody Sha31 (epitope YEDRYYRE). To control for PrP^res^ isoform, MM1 sCJD (type 1) and VV2 sCJD (type 2) isolates were included in each gel. The PrP^res^ isoform results for each passage and mouse line are summarized in Table 1

Tissue homogenates (10% w/v) of cerebral cortex (frontal, parietal, or temporal) from each case were subjected to Western Blot (WB) analysis to determine the PrP^res^ banding patterns (Table 1, Fig. 1). All E200K-Met_129_/Met_129_ and E200K-Met_129_/E200K-Met_129_ cases displayed a PrP^res^ type 1 profile, while the E200K-Met_129_/Val_129_ cases exhibited either type 1, type 2, or mixed type 1/type 2 profiles. The single E200K-Val_129_/Val_129_ case showed a type 2 PrP^res^ profile (Fig. 1). These findings align with previously published data [20, 27].

### Bioassays transmission results

The brain homogenates were also inoculated into transgenic mice expressing human PrP homozygous for methionine (tgMet) or valine (tgVal) at codon 129, as these mouse models express approximately four times more PrP^C^ than human brain tissue [11]. Control cases of sCJD from France (*n* = 7) and the UK (*n* = 4), classified as MM1, MV1, MV2, and VV2, were also transmitted to tgMet and tgVal mice (Supplementary Table S2). Previous studies using these cases identified two sCJD strains, M1^CJD^ and V2^CJD^, which were present either as pure components or as a mixture [10].

For the E200K gCJD cases, three distinct transmission patterns were observed.

**Fig. 2:**
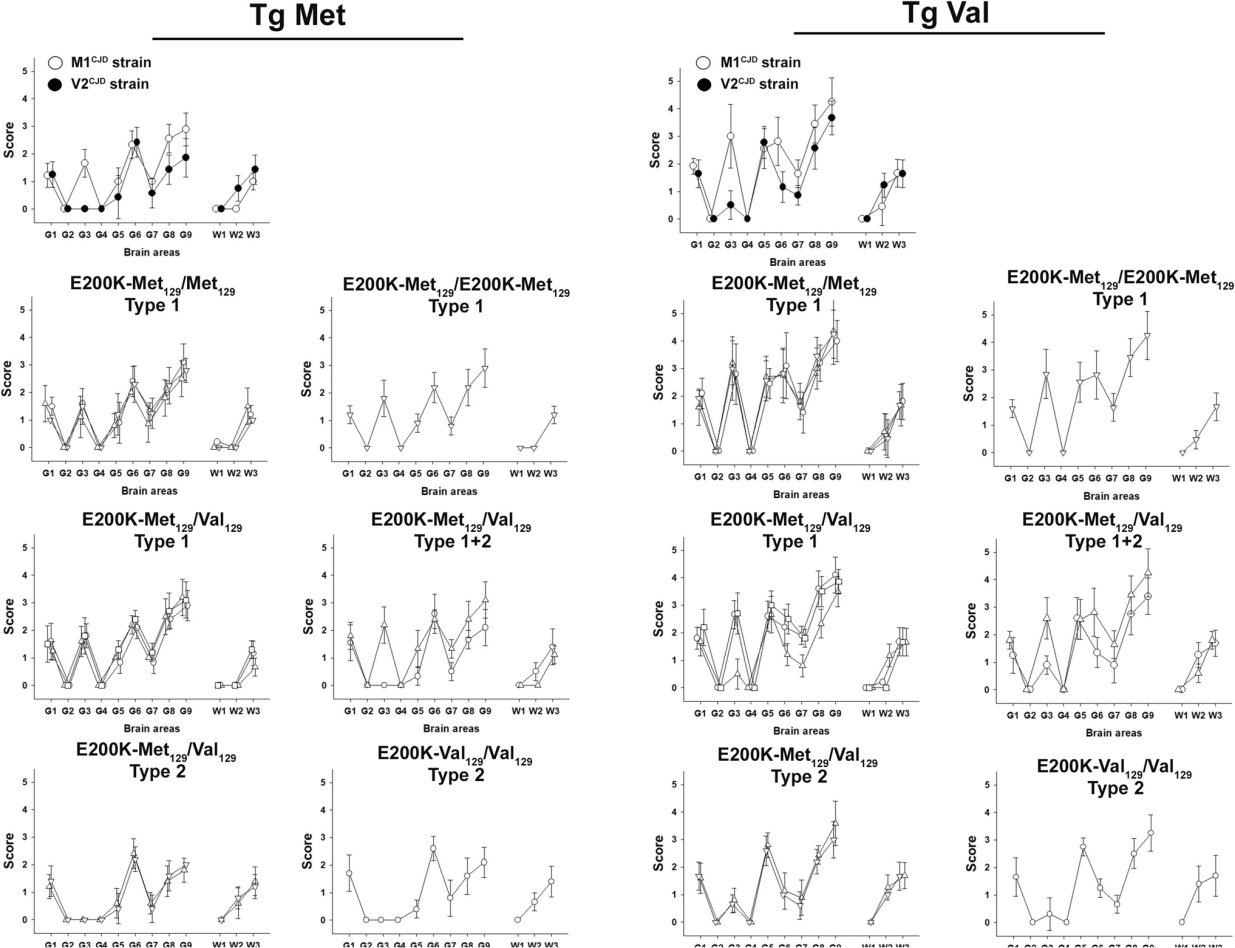
Vacuolar lesion profiles in the brain of human PrP-expressing mice (tgHu) inoculated with E200K gCJD isolates. E200K gCJD isolates from patients in France, Slovakia, and Spain (see Table 1) were inoculated intracerebrally (20µL per mouse) into transgenic mice expressing human PrP with Met_129_ (tgMet) or Val_129_ (tgVal) (n = 6 per group). After two serial passages, standardized vacuolar lesion profiles were established in the brains of the mice. For comparison, lesion profiles corresponding to the M1^CJD^ (О) and V2^CJD^ (●) strains are presented. Results are shown according to the patients'genotype and PrPres Western blot profiles of the original isolates (see Table 1). Brain regions are as follows: G1: medulla oblongata, G2: cerebellar cortex, G3: superior colliculus, G4: hypothalamus, G5: thalamus, G6: hippocampus, G7: septum, G8: cerebral cortex at the level of the thalamus, G9: frontal cerebral cortex, W1: cerebellar white matter, W2: mesencephalic tegmentum, W3: cerebral peduncles. E200K-Met_129_/Met_129_ Type 1 PrP^res^: cases 1 (▽), case 2 (△) and case 8 (О). E200K-Met_129_/E200K-Met_129_ Type 1 PrP^res^: case 15(∇). E200K-Met_129_/Val_129_ Type 1 PrP^res^: case 16 (△), case 19 (□), case 20 (О). E200K-Met_129_/Val_129_ Type 1 + 2 PrP^res^: case 18 (△), case 22 (О). E200K-Met_129_/Val_129_ Type 2 PrP^res^: case 17 (△), case 21 (▽). E200K-Val_129_/Val_129_ type 2 PrP^res^: case 24 (О)

The most frequent pattern was observed in all E200K-Met_129_/Met_129_ cases (cases 1–14), the single E200K-Met_129_/E200K-Met_129_ case (case 15), and some E200K-Met_129_/Val_129_ cases (cases 18, 19, and 20). These cases exhibited short incubation periods in tgMet (~ 200 days post-inoculation [dpi]) and longer periods in tgVal (~ 300 dpi) (Table 1). A type 1 PrP^res^ banding pattern was observed in both mouse models. This pattern matched the transmission characteristics of the M1^CJD^ strain, typically seen in MM1 and MV1 sCJD patients (Supplementary Table S2). Vacuolar lesion profiles in the brains of mice after the second passage confirmed the propagation of the M1^CJD^ strain (Fig. 2).

The second pattern was observed in some E200K-Met_129_/Val_129_ cases (cases 17, 21, 22, and 23) and the single E200K-Val_129_/Val_129_ case (case 24). It was characterized by shorter incubation periods in tgVal (~ 160–180 dpi) and longer ones in tgMet (~ 400–700 dpi) (Table 1, Fig. 1). Type 1 PrP^res^ accumulated in tgMet, while type 2 PrP^res^ was detected in tgVal. This pattern resembled V2^CJD^ strain transmission, observed in French and UK MV2 and VV2 sCJD cases (Table 2). The lesion profiles confirmed the propagation of the V2^CJD^ strain (Fig. 2).

**Table 2:** Transmission into human PrP Met_129_-expressing mice and RT-QuIC end-point titration of E200K gCJD isolates (frontal cortex and peripheral tissue homogenates)

Case	Tissue	Passage 1			Passage 2			RT-QuIC Seeding activity titres (log_10_SD_50_/mg of tissue)
		n/no	Incub (mean ± SD)	PrP^res^ type	n/no	Incub (mean ± SD)	PrPres type	
3	Frontal cortex	6/6	194 ± 8	1	6/6	193 ± 8	1	13.45 ± 0.49
	Spleen	3/6	263, 406, 556	1	6/6	254 ± 13	1	8.95 ± 0.75
5	Frontal cortex	6/6	193 ± 9	1	6/6	204 ± 11	1	13.2 ± 0.69
	Spleen	6/6	263 ± 15	1	6/6	208 ± 7	1	10.2 ± 0.69
6	Frontal cortex	6/6	205 ± 4	1	6/6	202 ± 7	1	13.45 ± 0.49
	Spleen	0/6	> 700	–	ND			neg*
	Liver	0/6	> 700	–	ND			6.82 ± 0.36
	Lymph node	0/6	> 700	–	ND			7.2 ± 0
	Lung	0/6	> 700	–	ND			6.57 ± 0.36
	Heart	0/6	> 700	–	ND			neg*
	Kidney	5/6	327 ± 54		6/6	205 ± 9	1	7.95 ± 0.49
7	Frontal cortex	6/6	232 ± 6	1	6/6	209 ± 9	1	12.95 ± 0.49
	Spleen	2/6	317, 323	1	6/6	212 ± 7	1	9.7 ± 0.57
	Liver	0/6	> 700	–	ND			7.45 ± 0.49
	Lymph node	1/6	330	1	6/6	204 ± 6	1	7.7 ± 0.57
	Lung	1/6	353	1	6/6	241 ± 17	1	7.45 ± 0.49
	Heart	5/5	360 ± 72		6/6	198 ± 4	1	9.45 ± 0.0.49
	Kidney	0/6	> 700	–	ND			neg*
21	Frontal cortex	6/6	525 ± 67	1	6/6	559 ± 42	1	13.45 ± 0.49
	Spleen	0/6	> 700	–	ND			7.95 ± 0.49

Transgenic mice that express human Met_129_ PrP (tgMet) were inoculated intracerebrally (20 µL per mouse) with frontal cortex or peripheral tissues homogenates (10% weight/volume) collected postmortem in E200K-Met_129_/Met_129_ (cases 3, 5, 6, and 7) and E200K-Val_129_/Val_129_ (case 21) Creutzfeldt–Jakob (CJD) affected patients originating from Slovakia. Data from these four cases are also reported in Table 1. After the first passage, brain from clinically affected mice were pooled and used for a second passage in the same line. The PrP^res^ WB isoforms (type 1 or type 2) identified in mouse brains are reported for each two passages. n/n0: number of diseased/number of inoculated mice. Incubation periods (time to death in days) are shown as mean ± standard deviation (SD) or (when attack rate was ≤ to 50%) as individual incubation period. *ND* not done. Seeding activity titres (provided as log_10_ SD_50_/mg of tissue ± standard deviation) were estimated by RT-QuIC using the Spearman–Kärber limiting dilution method. The Poisson’s probabilistic model was applied when less than 100% positive reactions were observed in the first tested dilution [8]. *: All tested replicates (*n* = 4) of the first tested dilution (10^–2^ of the original isolate) were negative

A third transmission pattern was observed in one E200K-Met_129_/Val_129_ case (case 16). This was characterized by short incubation periods in both tgMet (~ 200–300 dpi) and tgVal (~ 160–200 dpi) (Table 1). Type 1 PrP^res^ was observed in tgMet, while type 2 PrP^res^ accumulated in tgVal (Fig. 1). The lesion profiles in tgMet matched those of M1^CJD^, while tgVal corresponded to V2^CJD^ (Fig. 2). This pattern mirrored transmission results from artificial mixtures of cloned M1^CJD^ and V2^CJD^ strains (Supplementary Table S3, Fig. 2 and reference [10]).

These findings strongly suggest that the prion strains in E200K gCJD patients are highly similar, if not identical, to those found in sCJD cases, based on incubation periods, biochemical signatures, and lesion profiles after iterative passages in transgenic models.

### Peripheral tissue analysis

We next investigated prion infectivity in the peripheral tissues of four E200K-Met_129_/Met_129_ CJD patients (cases 3, 5, 6, 7) and one E200K-Met_129_/Val_129_ CJD patient (cases 21) (Table 2). The brain material from these patients exhibited either consistent M1^CJD^ (cases 3, 5, 6, 7) or V2^CJD^ (case 21) transmission patterns in tgMet and tgVal mice.

**Fig. 3:**
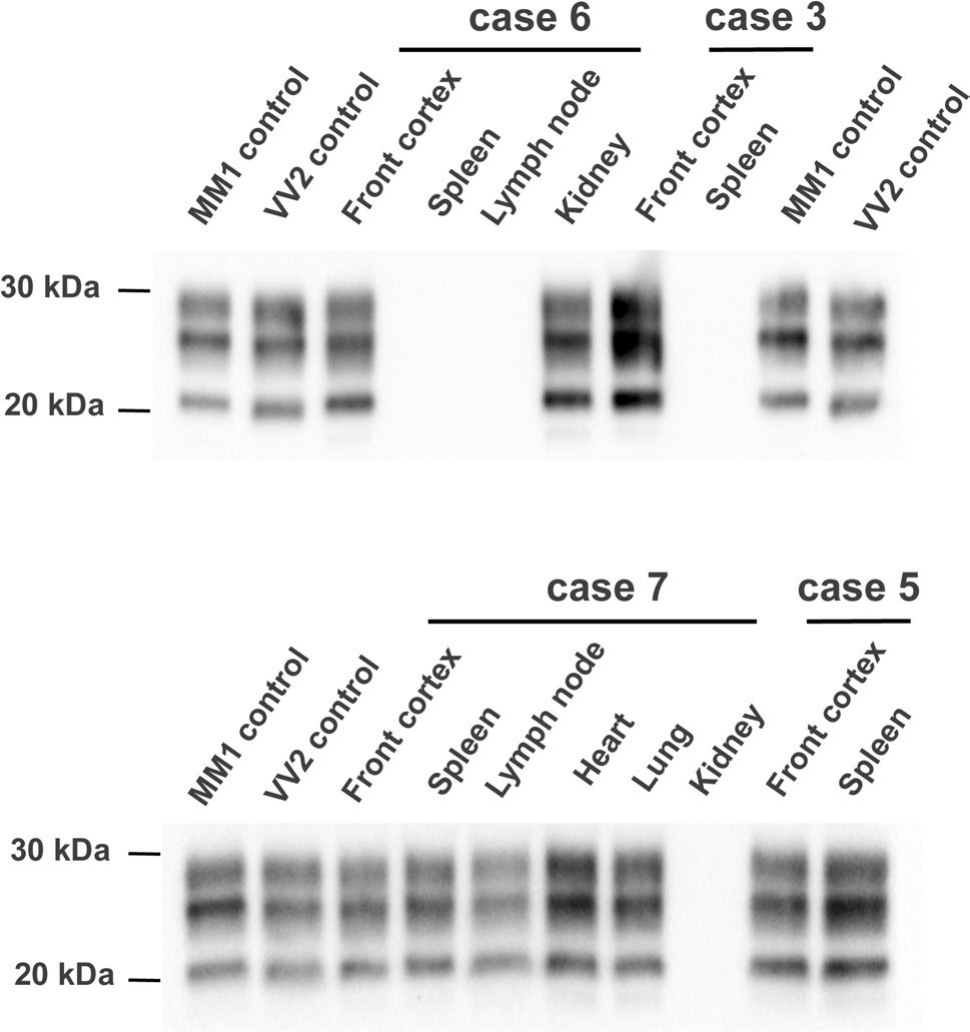
PrP^res^ western blot in the brains of tgMet mice inoculated with brain and peripheral tissues from E200K gCJD Patients. Western blot analysis of proteinase K-resistant prion protein (PrP^res^) was conducted on brain homogenates from transgenic mice expressing human PrP with Met_129_ (tgMet) after two serial intracerebral passages (n = 6 per group). The mice were inoculated with 20µL of a 10% homogenate prepared from brain or peripheral tissues of E200K gCJD patients (refer to Tables 1 and 2). PrP^res^ was detected using the anti-PrP monoclonal antibody Sha31, targeting the YEDRYYRE epitope. For comparison, MM1 sCJD (type 1) and VV2 sCJD (type 2) prion isolates were included as controls. PrP^res^ isoform identification results for each passage are summarized in Table 2

**Fig. 4:**
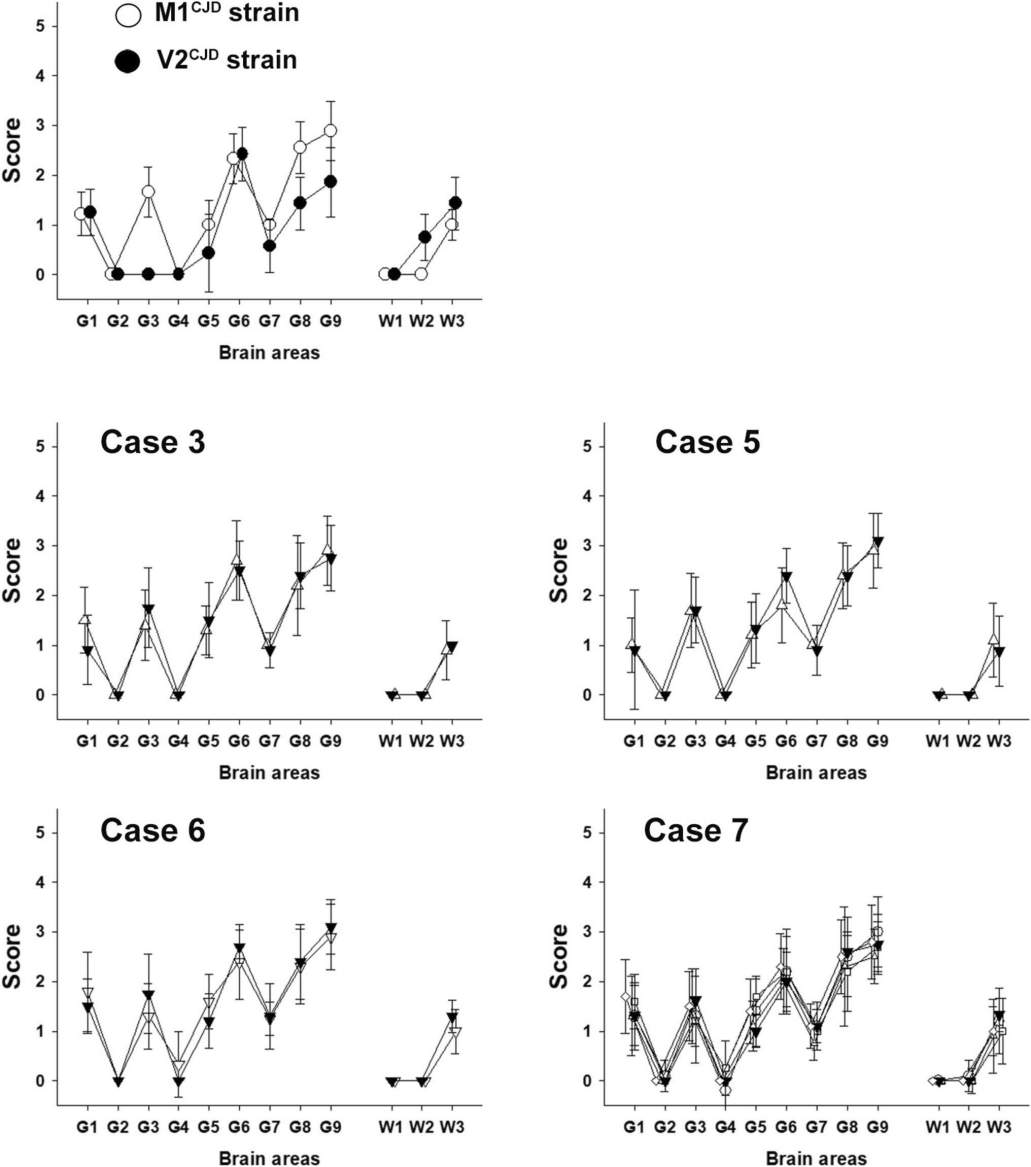
Vacuolar lesion profile in the brains of tgMet mice inoculated with brain and peripheral tissue homogenates from E200K. gCJD patients standardized vacuolar lesion profiles were established in the brains of tgMet mice after two serial intracerebral passages (*n* = 6 per group). The mice were inoculated with 20µL of a 10% homogenate prepared from either brain or peripheral tissues of E200K gCJD patients (refer to Tables 1 and 2). Brain regions are as follows: G1: medulla oblongata, G2: cerebellar cortex, G3: superior colliculus, G4: hypothalamus, G5: thalamus, G6: hippocampus, G7: septum, G8: cerebral cortex at the level of the thalamus, G9: frontal cerebral cortex, W1: cerebellar white matter, W2: mesencephalic tegmentum, W3: cerebral peduncles. The lesion profiles from different tissue sources are indicated as follows: frontal cortex (▼), spleen (△), lymph node (◊), lung (О), heart (□). For comparison, lesion profiles corresponding to the M1^CJD^ (О) and V2^CJD^ (●) strains are presented

Peripheral tissue homogenates (10%) from spleen, lymph node, heart, lung, and kidney were inoculated into tgMet mice. Transmission was detected in at least one tissue per patient, with positive results in all tissue categories except liver (Table 2). Second passage of positive isolates in tgMet resulted in 100% attack rates, with incubation periods ranging from 190 to 240 days. The PrP^res^ banding pattern (Fig. 3) and vacuolar lesion profiles in tgMet inoculated with peripheral tissues (Fig. 4) were identical to those observed with brain homogenates, confirming the same prion strain was present in both CNS and peripheral tissues. No transmission was observed in control tissues from non-CJD patients.

### Prion seeding activity in peripheral tissues

**Fig. 5:**
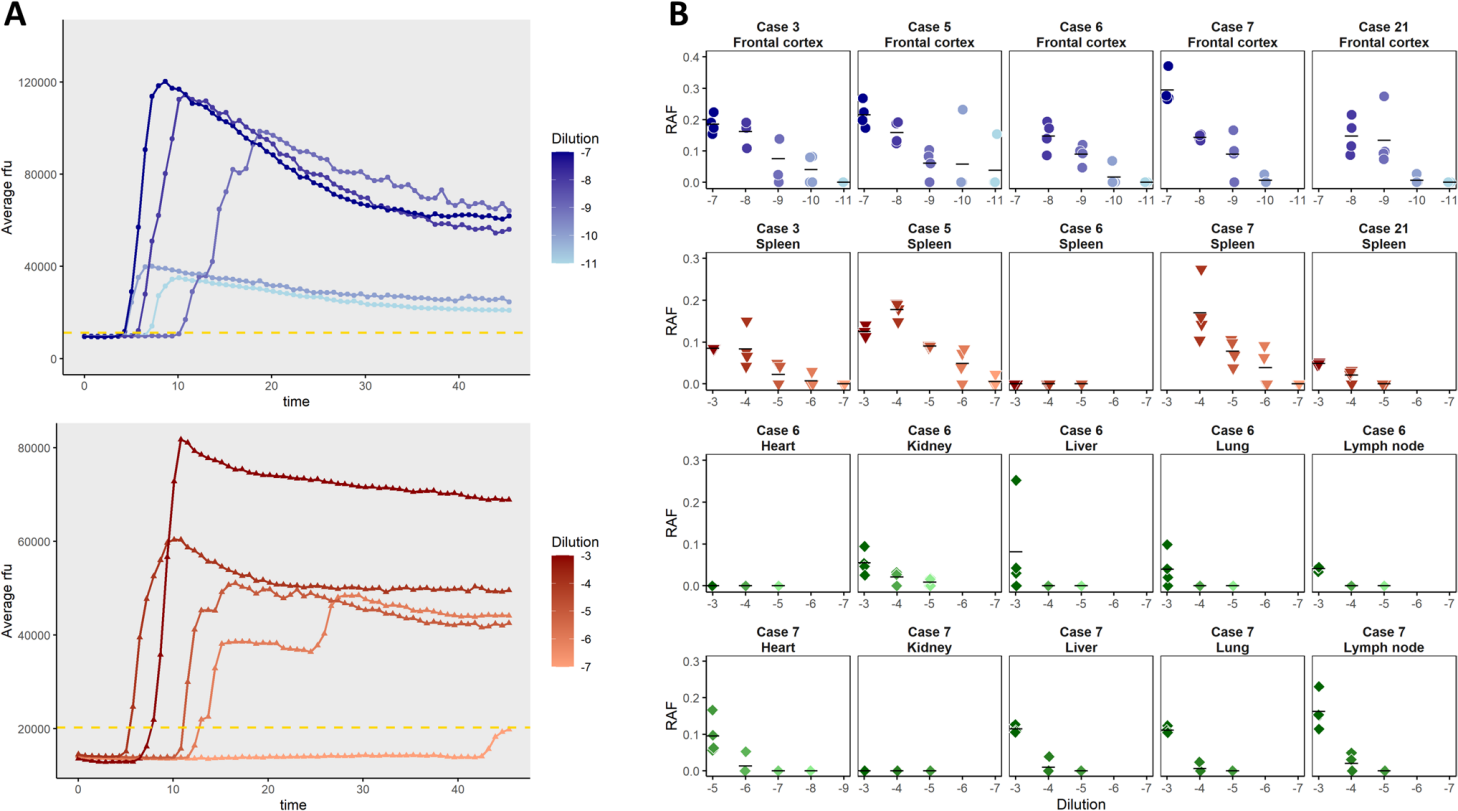
Real-time quaking-induced conversion analysis of brain and peripheral tissue homogenates from E200K gCJD patients. **A** Representative RT-QuIC amplification curves from brain (case 5, upper panel) and spleen (case 5, lower panel). The average fluorescence (given as relative fluorescence units or rfu) of all technical replicates at each time point is displayed. Each dilution is displayed in a different color. The yellow dotted line represents the threshold above which a sample is considered positive, calculated as the average fluorescence of the plate at time = 0 plus three times the standard deviation. **B** Rate of amyloid formation (RAF) values (calculated as the inverse of the time to threshold) per dilution for each tested tissue and case. Each dot represents a single technical replicate, and the bar represents the mean of the four technical replicates. Replicates with an RAF value > 0 are positive

To quantify prion levels in peripheral tissues, we applied real-time quaking-induced conversion (RT-QuIC) to both nervous and peripheral tissues from E200K gCJD patients. Seeding activity (SA) was detected in all tissues that tested positive by bioassay (Table 2, Fig. 5). SA titres were highest in brain samples (~ 13 log_10_ SD_50_ per mg), consistent with high attack rates and short incubation periods. Peripheral tissue SA titres were lower (ranging from 6.57 to 10.2 log_10_ SD_50_ per mg) but detectable even in tissues without positive bioassay results, indicating the higher sensitivity of RT-QuIC compared to classical bioassay for detecting human gCJD prions.

## Discussion

### Representativeness of the E200K gCJD case panel

Bioassays in animal models remain the gold standard for prion strain characterization, enabling the phenotyping of propagated prions based on vacuolar lesion profiles and incubation periods.

However, research focusing on the prion strain typing of E200K-associated Creutzfeldt–Jakob disease (CJD) has been fragmented and limited [7, 24, 37, 47].

In this study, we present the first comprehensive attempt to establish the prion strains responsible for E200K genetic CJD (gCJD) by analyzing 24 cases from France, Spain, and Slovakia using human PrP-expressing transgenic mice (tgMet and tgVal).

While our goal was to capture the global diversity of prion strains in E200K gCJD patients, practical limitations, such as the high cost and long duration of bioassays, and the rarity of E200K-Val_129_ cases, constrained the number of cases we could include. Furthermore, due to the limited availability of biological material, we were unable to bioassay different brain regions from each patient. Previous research has shown that sCJD patients can harbor multiple prion strains or strain mixtures in different brain regions, which may also apply to gCJD cases. This was not fully explored in our study due to material constraints.

Despite these limitations, our results clearly demonstrate the presence of two distinct prion strains, either alone or as mixtures, in the E200K gCJD cases. Given the number of cases (*n* = 22) and their geographical diversity, it is reasonable to conclude that these two strains likely represent the dominant prion variants in E200K gCJD. However, we cannot exclude the possibility that other prion strains may be implicated in some cases, particularly given the limitations in brain region sampling.

### Prion strains in E200K gCJD patients

The prion strains identified in E200K gCJD brain homogenates following transmission into tgHu mouse models were identical to those previously found in sCJD patients using the same methodology [10]. Specifically, the strains correspond to M1^CJD^ and V2^CJD^, which are responsible for the most common clinico-pathological forms of sCJD, predominantly observed in MM/MV1 and VV/MV2 patients, respectively [6, 10, 11, 23].

Of note, the correlation between prion strain type and *PRNP* codon 129 polymorphism in E200K gCJD closely mirrors that seen in sCJD. In both diseases, patients homozygous for methionine at codon 129 (Met_129_/Met_129_) harbor the M1^CJD^ strain, while heterozygous patients (Met_129_/Val_129_) can present with either the M1^CJD^ or V2^CJD^ strains, or a mixture of both. In our study, this was evident in the E200K-Met_129_/Val_129_ cases, which showed a mixture of prion strains following transmission in tgHu mice.

Although we had only one case of E200K-Val_129_/Val_129_, the presence of the V2^CJD^ strain in this case is consistent with strain-typing studies of sCJD cases homozygous for valine at codon 129, where V2^CJD^ is typically found in Val_129_/Val_129_ patients with PrP^res^ type 2 in the brain. While further studies are needed to confirm this observation, it suggests that the *PRNP* codon 129 polymorphism plays a similar role in strain selection in both gCJD and sCJD.

The apparent identity of strains between sCJD and gCJD E200K that we observed is in line with the previous transmission studies in voles showing that E200K gCJD produce disease phenotypes indistinguishable from those of sCJD type 1 [37]. It also agrees with the fact that genetic CJD forms share common neuropathological traits with sporadic cases, particularly a classification in very similar histotypes, which depend on the polymorphism at codon 129 of *PRNP* and the biochemical PrP^res^ type [4], although some differences exist, mainly at the level of immunohistochemical PrP deposition patterns [27]. Whether these variations can be explained by the presence of different minor strain components in gCJD brains or by other factors remains an open and interesting question.

### Role for a somatic *PRNP* E200K mutation in sporadic CJD

Sporadic Creutzfeldt–Jakob disease (sCJD) remains the most common form of human prion disease, but despite extensive research its underlying cause still remains unclear. It is uncertain whether sCJD originates from endogenous factors, such as the spontaneous misfolding of normal cellular prion protein (PrP^C^), or from exposure to an unidentified exogenous agent. Numerous case–control studies have explored various risk factors, including dietary habits, occupational exposure to animal products, and prior medical procedures. While some studies have suggested links to surgeries and blood transfusions, the evidence has been inconsistent and unable to establish definitive associations [13, 14, 35, 36, 50].

The most widely accepted hypothesis suggests that sCJD results from a random misfolding event in PrP^C^ or a somatic mutation in the *PRNP* gene [44], analogous to spontaneous mutations observed in other neurodegenerative diseases, such as Alzheimer’s disease, Parkinson’s disease, and amyotrophic lateral sclerosis (ALS). Somatic mutations in genes linked to these diseases are increasingly identified in the brains of affected patients [29, 31, 42], suggesting a similar mechanism could underlie sCJD.

Some authors have hypothesized that the instability of the octapeptide repeat region of the PrP^C^ protein may lead to the accumulation of somatic mutations over the lifetime, eventually initiating PrP^Sc^ formation and triggering disease [30]. Post-zygotic mutation D178N was confirmed as the cause of disease in a patient showing somatic mosaicism with negative family history [1]. Moreover, a recent study by Won et al. (2023) found a higher frequency of the E200K mutation in hippocampal and frontal cortex samples from sCJD cases compared to age- and genotype-matched controls, specifically in Met_129_/Met_129_ and Met_129_/Val_129_ variants [53]. However, contrasting evidence from McDonough et al. (2024) found no evidence of somatic *PRNP* mutations in neuropathologically affected brain areas from sCJD patients, as revealed by deep DNA sequencing [33].

The identification of the same dominant prion strains (M1^CJD^ and V2^CJD^) in both sCJD and familial E200K gCJD patients provides an indirect argument supporting the hypothesis that somatic mutations in *PRNP* could be a key driver in the occurrence of sporadic Creutzfeldt-Jakob disease (sCJD). However, this hypothesis remains challenging to substantiate. Efforts to model heritable prion diseases in transgenic (Tg) mice expressing *PRNP* mutations that are strongly linked to genetic forms of Creutzfeldt–Jakob disease (gCJD) in humans have met with only limited success [51]. While transgenic mice expressing either the human E200K PrP variant or the mouse equivalent mutation (E199K) have been developed, these models have consistently failed to spontaneously develop an authentic prion disease [3, 7, 19, 22, 48].

Bringing definitive evidence of the role of the E200K mutation in the occurrence of sporadic Creutzfeldt–Jakob disease (sCJD) will therefore require further extensive investigation. This will likely include advanced techniques, such as single-cell sequencing to detect low-frequency somatic mutations, longitudinal studies of brain tissue, and more sophisticated animal models that better replicate the spontaneous development of prion diseases.

### E200K as a proxy to study preclinical sCJD

Our findings, which reveal a clear overlap in prion strains between E200K gCJD and sCJD, along with comparable prion seeding activity and infectivity patterns in peripheral tissues, strongly indicate that these two conditions share significant pathological similarities. This positions E200K gCJD as a highly informative model for studying the early stages of sCJD pathogenesis, potentially offering critical insights that could enhance both the understanding and clinical management of sporadic CJD cases.

Given the rare incidence of sCJD in the general population, it is challenging to conduct prospective studies on individuals prior to disease onset. However, the high penetrance of the E200K mutation allows for longitudinal studies in carriers, offering an opportunity to explore the preclinical phase of disease development. This could facilitate the identification of early biomarkers and the development of diagnostic tests that may eventually benefit both at-risk individuals and the broader population.

The age at disease onset and clinical progression in sCJD are strongly influenced by disease subtype [41]. Our finding that prion strains found in E200K gCJD cases align with disease subtypes in a manner mirroring patterns seen in sCJD could further aid in refining prognostic tools for patients, ensuring a more personalized approach to disease management.

Additionally, animal model studies have clearly demonstrated that the efficacy of anti-prion treatments is heavily influenced by both the timing of therapeutic intervention and the specific prion strain involved. In this context, conducting therapeutic trials in E200K mutation carriers, who are at higher genetic risk, could not only provide direct benefits to these individuals but also offer crucial data for optimizing treatment strategies for sCJD patients. By understanding the timing and strain-specific effects of potential therapies, these trials could inform the best practices for treatment in sporadic cases as well.

The observed similarities in prion strains between E200K gCJD and sCJD emphasize the need for continued research into strain diversity and its value in the understanding of prion diseases. Further studies involving *PRNP* mutation carriers with a high risk of developing CJD could offer essential guidance for developing targeted therapies and improving care for those affected by these devastating diseases.

## Supplementary Information

Below is the link to the electronic supplementary material.

Supplementary file1 (PDF 122 KB)
