# Supplementary material for: Characterization of prion strains and peripheral prion infectivity patterns in E200K genetic CJD patients
Source: Acta Neuropathol. 2025 Jun 16;149(1):62. doi: 10.1007/s00401-025-02903-5 (PMC12170713; doi:10.1007/s00401-025-02903-5)
Supplement: Supplementary file 1 — Supplementary file1 (PDF 122 KB) [file 401_2025_2903_MOESM1_ESM.pdf]

Supplementary Table S1: Biochemical typing of several brain areas (frontal and parietal cortices and cerebellum) of E200K gCJD patients.

Isolates							
**Case**	**Genotype**	**Orig.**	**Age**	**Sex**	**Frontal cortex**	**Parietal cortex**	**Cerebellum**
**1**	E200K-Met$_{129}$/Met$_{129}$	**Fr**	F	64	**1**	**1**	**1**
**2**		**Slov**	F	52	**1**	**NA**	**NA**
**3**		**Slov**	M	67	**1**	**NA**	**NA**
**4**		**Slov**	F	73	**1**	**NA**	**NA**
**5**		**Slov**	F	52	**1**	**NA**	**NA**
**6**		**Slov**	F	60	**1**	**1**	**NA**
**7**		**Slov**	F	65	**1**	**1**	**NA**
**8**		**Sp**	F	65	**1**	**1**	**1**
**9**		**Sp**	M	30	**1**	**1**	**1**
**10**		**Sp**	M	50	**1**	**1**	**1**
**11**		**Sp**	F	50	**1**	**1**	**1**
**12**		**Sp**	M	64	**1**	**1**	**1**
**13**		**Sp**	M	58	**1**	**1**	**1**
**14**		**Fr**	M	53	**1**	**1**	**1**
**15**	E200K-Met$_{129}$/E200K Met$_{129}$	**Slov**	F	29	**1**	**NA**	**NA**
**16**	E200K-Met$_{129}$/Val$_{129}$	**Fr**	M	58	**1**	**1+2**	**1+2**
**17**		**Slov**	F	64	**2**	**NA**	**NA**
**18**		**Slov**	F	65	**1+2**	**1**	**NA**
**19**		**Slov**	M	53	**1**	**1**	**NA**
**20**		**Slov**	F	55	**1**	**1**	**NA**
**21**		**Slov**	F	71	**2**	**2**	**NA**
**22**		**Sp**	F	65	**1+2**	**NA**	**NA**
**23**		**Sp**	M	62	**1+2**	**NA**	**NA**
**24**	E200K-Val$_{129}$/Val$_{129}$	**Sp**	F	53	**2**	**2**	**2**

Homogenates prepared from the different brain areas of E200K gCJD patients originating (orig.) from three different countries (France (Fr), Slovakia (Slov), or Spain (Sp)) were subjected to proteinase K (PK) digestion followed by Western blotting as previously described [28]. The age and sex (F: female, M: male) of the patients are indicated. Immunodetection was carried on PVDF membranes out using the monoclonal primary antibody Sha31 (1 µg/mL), which recognizes amino acids 145-152 (YEDRYYRE) of PrP, and an anti-mouse HRP-conjugated secondary antibody (Biorad) [16]. This method allowed the identification of distinct PrP^{res} banding patterns including type 1 (21 kDa), type 2 (19 kD), an intermediate type (i) and mixes of types 1+ 2 . NA: tissue not available.

Supplementary Table S2: Transmission of sporadic CJD isolates (10% brain homogenates) into mice expressing the human PrP (methionine, valine, at codon 129).

					TgMet129				TgVal129				
					Passage 1		Passage 2		Passage 1		Passage 2		
**Isolate number**	**Codon 129**	**PrP^{res} type**	**origin**	**Brain area**	**Survival time**	**PrP^{res} type**	**Survival time**	**PrP^{res} type**	**Survival time**	**PrP^{res} type**	**Survival time**	**PrP^{res} type**	**Identified strain(s)**
**25**	**MM**	**1**	**Fr**	**Frontal cortex**	230±13	1	201±11	1	336±8	1	284±5	1	**M1^{CJD}**
**26**	**MM**	**1**	**UK**	**Frontal cortex**	226±4	1	ND		326±9		ND		**M1^{CJD}**
**27**	**MV**	**1**	**Fr**	**Frontal cortex**	276±19	1	205±7	1	296±13	1	282±9	1	**M1^{CJD}**
**28**	**MV**	**1**	**UK**	**Frontal cortex**	197±6	1	209±17	1	289±13	1	289±8	1	**M1^{CJD}**
**29**	**MV**	**1**	**Fr**	**Frontal cortex**	205±16	1	220±13	1	232±62	2	172±12	2	**M1^{CJD}+V2^{CJD}**
**30**	**MV**	**2**	**UK**	**Frontal cortex**	470±23	1	211±12	1	184±9	2	180±6	2	**M1^{CJD}+V2^{CJD}**
**31**	**MV**	**2**	**Fr**	**Frontal cortex**	375±45	1	205±5	1	184±9	2	180±6	2	**M1^{CJD}+V2^{CJD}**
**32**	**MV**	**2**	**UK**	**Frontal cortex**	578±28	1	492±70	1	219±17	2	174±3	2	**V2^{CJD}**
**33**	**MV**	**2**	**Fr**	**Caudate**	518±84	1	571±14	1	180±8	2	161±8	2	**V2^{CJD}**
**34**	**VV**	**2**	**Fr**	**Frontal cortex**	626±85	1	548±24	1	198±7	2	170±7	2	**V2^{CJD}**
**35**	**VV**	**2**	**Fr**	**Frontal cortex**	521±65	1	216±1	1	188±13	2	166±6	2	**M1^{CJD}+V2^{CJD}**

Transgenic mice that express the Met_{129} (tgMet), Val_{129} (tgVal) human PrP were inoculated intra-cerebrally (20µL per mouse) with sporadic Creutzfeldt-Jakob (sCJD) brain tissue homogenates (frontal cortex or caudate nucleus) from patients originating (orig.) from France (Fr) or the United Kingdom (UK). The sCJD patients displayed different *PRNP* genotypes at codon 129 (MM: homozygous Met_{129}, VV: homozygous Val_{129}, MV: heterozygous Met/Val_{129}) and PrP^{res} Western blot isoforms (type 1 or type 2). After the first passage, brain tissue from clinically affected mice were pooled and used for a second passage in the same line. The PrP^{res} WB isoforms (type 1 or type 2) identified in mouse brains are reported for each two passages. Survival times are shown as mean ± standard deviation (SD). 100% attack rate transmission were observed in all cases. ND: not done. With the exception of isolate 26, transmission data have already been use in a previous publication. The prion strain(s) identified in each isolate (strain typing based on survival time and vacuolar lesion profile in the brain) are indicated in the table. For full data (lesion profile data and WB PrP^{res} typing) please refer to Cassard et al 2020 [10].

Supplementary Table S3: Bioassay transmission in tg Hu mice of artificial V2^{CJD} / M1^{CJD} strains mixture

Artificial strain Mixture composition		TgMet$_{129}$						TgVal$_{129}$					
		Passage 1			Passage 2			Passage 1			Passage 2		
		n/no	Survival time (mean±SD)	PrP^{res} type	n/no	Survival time (mean±SD)	PrP^{res} type	n/no	Survival time (mean±SD)	PrP^{res} type	n/no	Survival time (mean±SD)	PrP^{res} type
**V2 neat**	**M1 neat**	6/6	207±3	1	6/6	205±2	1	6/6	173±7	2	6/6	179±1	2
**V2 neat**	**-**	6/6	549±65	1	6/6	506±17	1	6/6	175±5	2	6/6	174±3	2
**M1 neat**	**-**	6/6	210±12	1	6/6	199±3	1	6/6	295±5	1	6/6	283±11	1
**V2 neat**	**M1 10^{-1}**	6/6	244±11	1	6/6	203±8	1	6/6	177±6	2		ND	
**V2 neat**	**M1 10^{-2}**	6/6	250±5	1	6/6	198±3	1	6/6	171±6	2		ND	
**V2 neat**	**M1 10^{-3}**	6/6	329±89	1	6/6	207±5	1	6/6	173±10	2		ND	
**V2 neat**	**M1 10^{-4}**	6/6	463±67	1	6/6	201±7	1	6/6	176±10	2		ND	
**V2 neat**	**M1 10^{-5}**	6/6	513±32	1	6/6	505±24	1	6/6	176±5	2		ND	
**M1 neat**	**V2 10^{-1}**	6/6	207±10	1		ND		6/6	190±8	2	6/6	179±3	2
**M1 neat**	**V2 10^{-2}**	6/6	211±9	1		ND		6/6	233±13	2	6/6	189±8	2
**M1 neat**	**V2 10^{-3}**	6/6	199±9	1		ND		6/6	245±2	2	6/6	189±8	2
**M1 neat**	**V2 10^{-4}**	6/6	203±8	1		ND		6/6	288±2	1	6/6	229*<br>266, 267, 287, 288,299	2<br>1
**M1 neat**	**V2 10^{-5}**	6/6	202±8	1		ND		6/6	283±8	1	6/6	289±5	1

M1^{CJD} and V2^{CJD} strains were obtained by the endpoint titration of a MM1 and VV2 sCJD isolate in Met$_{129}$ (tgMet) or Val$_{129}$ (tgVal) human PrP-expressing mice respectively (see supplementary table 1). Brains from tgMet (inoculated with M1^{CJD} strain) and tgVal (inoculated with V2^{CJD} strain) were used to produce stock solutions (10% tissue homogenates). 1/10 dilution series (in phosphate buffer saline) of each stock solution were prepared. M1^{CJD}/V2^{CJD} strain mixtures were obtained by mixing equal volume of each component at the chosen dilutions. Samples were then transmitted (two iterative passages) to tgMet and tgVal (intra-cerebral route, 20µL per mouse). Brains from first passage positive mice (PrP^{res} presence in the brain) were pooled and used for the second passage. The PrP^{res} WB isoforms (type 1 or type 2) identified in mouse brains are reported. Survival times (time to death in days) are shown as mean ± standard deviation (SD). n/n0: number of diseased / number of inoculated mice. *: PrP^{res} WB profile of the mice in the group were not homogenous and the individual incubation periods of each animal are presented. ND: not done. These data were already used in a previously published study [10] .
